# The Private Nursing Home

**Published:** 1910-01-22

**Authors:** 


### THE PRIVATE NURSING HOME.

We have on more than one occasion hinted at the urgent desirability of subjecting all nursing homes to a system of inspection. The reasons for urging such an innovation, which would scarcely have universal approval, are obvious enough to those who know anything about the conditions under which private nursing is carried on in this country. What these conditions are, and what their existence means to the sick patient, we have already set forth in these columns, and we have no great wish to recapitulate the counts of our indictment against many of the private concerns that have sprung up within the last ten years. These concerns appeal to all classes of the public, professional as well as lay, and it is imperative that they should be up-to-date and thoroughly worthy of the trust that doctor and patient place in them. That trust, it may be worth while to observe, is purely a matter of faith in the majority of instances. The practitioner, especially the busy consultant who is professionally the greatest patron of these private homes, has no time to look closely into the management and hygienic conditions of the home; very often he does not possess the requisite knowledge to enable him to judge this management and these conditions properly. Still less capable of finding out the fallacies in the glowing prospectus that the home issues is the lay patient. Both medical attendant and patient, therefore, must, to a very large extent, take the claims of the private nursing home, as they must do those of the average hospital, on trust, and it is only proper that there should be some guarantee, official or semi-official, that this confidence is not abused by private nursing home superintendents.

In the Australian Colonies, it is interesting to see, the agitation for the compulsory inspection of private nursing homes at last promises to result in successful action by the State. The immediate cause of such action happens to be a particularly disgraceful incident which led to the death of a young woman from septicæmia in a "private hospital" at Melbourne. The coroner, at the inquest, pointed out the strong necessity of supervising these institutions, and the Government of Victoria has now drafted a bill providing for the systematic inspection of all private hospitals. Under this term are included all hospitals not receiving State or municipal aid, nursing homes and private clinics, and special hospitals belonging to brotherhoods or societies, and as an evidence of the wide field that the Act will cover, it may be mentioned that in the State of Victoria there are no fewer than 569 such institutions. Of these 161 are for medical and surgical cases, while 381 are reserved for the exclusive use of lying-in patients. The details of the new Act will be of interest to all hospital workers in view of the fact that legislation on similar lines appears to be highly desirable in this country. In Australia the necessity for such legislative action is, if anything, less urgent than here, for under the Health Act the Colonial Government is provided with plenary powers to deal with private hospitals. In this country, also, there exist legislative clauses which could be made to apply to nursing homes, but their interpretation is somewhat vague, and the Health Authority does not as yet fully understand its powers. At any rate, to put in motion these powers needs actual complaint, which can scarcely be forthcoming so far as the public is concerned.

The Australian Act provides, we understand, for the registration of all private hospitals. The term "private hospital" is strictly defined, the definition being broad enough to cover most of the anomalous institutions which can hardly be included under the title "nursing home." In these circumstances the Act gains in power and interest, as it will make provision for the regulation and registration of numerous "half-way" institutions, many of which are very much in want of proper supervision and detailed inspection. The Act comes into force this week, and under its terms owners or superintendents of private hospitals who have not been registered are liable to a fine not exceeding £50. Provision is made for a proper investigation into the methods of the institution before the registration license is granted or before its renewal. The Act appears to us to be a necessary one. It is a safeguard to the public and the profession alike, and it is high time that we in this country should attempt to imitate the progressive spirit that made its appearance possible upon the Australian Statute-book.

